# Social network analysis of a decade-long collaborative innovation network between hospitals and the biomedical industry in China

**DOI:** 10.1038/s41598-024-62082-3

**Published:** 2024-05-18

**Authors:** Xiang Liu, Hong Chen, Yue Liu, Jie Zou, Jiahe Tian, Tenzin Tsomo, Meina Li, Wenya Yu

**Affiliations:** 1 Affiliated Xihu Hospital, Hangzhou Medical College, Hangzhou, 310000 China; 2 Department of Respiratory Disease, The 903Rd Hospital of PLA, Hangzhou, 310000 China; 3 Shanghai Chest Hospital, Shanghai Jiao Tong University School of Medicine, Shanghai, 200030 China; 4 School of Public Health, Shanghai Jiao Tong University School of Medicine, Shanghai, 200025 China; 5 Department of Pharmacy, The 903Rd Hospital of PLA, Hangzhou, 310000 China; 6 Department of Military Medical Service, Faculty of Military Health Service, Naval Medical University, Shanghai, 200433 China

**Keywords:** Social network analysis, Collaborative innovation, Network, Hospital, Industry, Biomedicine, Health policy, Health services

## Abstract

Collaborative innovation between hospitals and biomedical enterprises is crucial for ensuring breakthroughs in their development. This study explores the structural characteristics and examines the main roles of associated key actors of collaborative innovation between hospitals and biomedical enterprises in China. Using the jointly owned patent data within the country’s healthcare industry, a decade-long collaborative innovation network between hospitals and biomedical enterprises in China was established and analyzed through social network analysis. The results revealed that the overall levels of collaborative innovation network density, collaborative frequency, and network connectivity were significantly low, especially in less-developed regions. In terms of actors with higher degree centrality, hospitals accounted for the majority, whereas a biomedical enterprise in Shenzhen had the highest degree centrality. Organizations in underdeveloped and northwest regions and small players were more likely to implement collaborative innovation. In conclusion, a collaborative innovation network between hospitals and biomedical enterprises in China demonstrated high dispersion and poor development levels. Stimulating organizations’ initiatives for collaborative innovation may enhance quality and quantity of such innovation. Policy support and economic investments, strategic collaborative help, and resource and partnership optimization, especially for small players and in less-developed and northwest regions, should be encouraged to enhance collaborative innovation between hospitals and the biomedical industry in China and other similar countries or regions.

## Introduction

The significance of collaborative innovation in the fields of healthcare and medicine has been well demonstrated^1^. Collaborative innovation is a large-span integrated organizational model, which should be carried out by multiple organizations (e.g., enterprises, governments, universities, healthcare institutions) to realize major scientific and technological innovation. Collaborative innovation is a new paradigm to break through the limitations of a single actor’s innovative capability, which has individually been unable to meet the demands for competitive advantages and economic growth^2^. Collaborative innovation can improve health outcomes, promote diagnosis and treatment approaches^3–5^, expand market values, maximize productivity, promote knowledge and resource sharing and exchange, and accelerate scientific and technological innovation^1,6,7^. In this approach, various organizations are encouraged to fully utilize their respective capabilities and advantages and integrate complementary resources under the guidance of, and according to mechanisms arranged by, the national government^8^. To realize such collaborative innovation and activate these advantages, the key is the strong development of various organizations (e.g., organizational vitality and capability)^9^. Collaborations within a single organization or between individuals have disadvantages due to limitations in capability and resource sharing and integration. Therefore, collaborative innovation between different organizations is of great significance. In China, the “Outline of the Healthy China 2030 Plan” in 2016 proposed that interdisciplinary collaboration should be the main model for the future development of medical scientific research^10^; collaboration between hospitals and enterprises has become an important model for innovative development^11^. In 2021, the “14th Five-Year Plan” for medical equipment industry development in China highlighted the principle of collaborative innovation between hospitals and enterprises and encouraged exploration of new collaborative innovation models^12^. Therefore, collaborative innovation between hospitals and the biomedical industry in China has been a crucial development direction.

In China, the rapid development of hospitals over recent years has resulted in high demand for innovation in clinical research, advancements in diagnosis and treatment approaches, and the transformation of medical fields. Moreover, the biomedical industry has entered a critical period of equal emphasis on research and development, transformation, and manufacturing. In 2010 and 2011, the Chinese government proposed policies that emphasized the importance of collaborative innovation between hospitals and biomedical enterprises for ensuring high-level hospital reforms and breakthroughs in the biomedical industry^13,14^. Therefore, since 2011, hospitals and biomedical enterprises in China have been experiencing rapid development and facing new opportunities and challenges. While the collaborative innovation between hospitals and the biomedical industry has been highlighted in China over the last ten years, little is known about whether a network system has been formed, and if so, the status of its development, successes, and failures. Additionally, more fundamental questions about such collaborations remain unclear, including identifying influential actors that accelerate collaborations and identifying potential new collaboration relationships^15^. This lack of clarity is closely related to the future development of both hospitals and the biomedical industry. Therefore, it is imperative to deeply explore the collaborative innovation network between hospitals and the biomedical industry.

Social network analysis (SNA) is an effective tool for analyzing and evaluating complex collaboration networks by studying the relationships and interactions between various social actors in a group^16^. SNA has been widely used to evaluate collaboration relationships in health care fields, which can help improve collaborations and identify influential actors who can facilitate collaborative relationships. Such SNA explorations primarily include collaborations in cancer care^17^, community health^18,19^, vaccine research^20^, nursing^21,22^, psychiatry^23^, medical education^24^, drug discovery and development^25^, scientific networks and organizations^15,16,26–29^, and scientific medical research^30–34^. These SNA studies provided evidence on collaboration features and patterns, and identified key points regarding improving collaboration levels and ranges. Therefore, SNA is a powerful tool that helps to deeply understand the collaborative innovation network between hospitals and the biomedical industry.

The collaborative innovation network reflects the teamwork referring to academic organizations, healthcare institutions, biomedical enterprises, the science community, and patients^26^. This highlights the vital role of the collaborative innovation relationship between hospitals and the biomedical industry. However, there is little research on the evaluation of the structure and relationships of the collaborative innovation network between hospitals and the biomedical industry using SNA. Additionally, in terms of SNA of the collaborative innovation network among other actors (except for hospitals and biomedical enterprises) mentioned above, measurements for evaluating the collaborative innovation network can be divided into two categories: publications and grants^15,20,35,36^ and self-reported collaborative relationships^21,26,29^. However, considering that the translation of research publications and grant achievements is one of the essential aims of collaborative innovation, the measurement for collaborative innovation network evaluation based on publications and grants is not appropriate. Therefore, an appropriate measure is urgently needed. Existing studies have indicated that collaborative patents is a significant form of collaborative innovation, which has a long tradition and has advantages when used to evaluate collaborative innovation^37^. Patents with joint ownership can accelerate the innovation process^37^, which further indicates the rationality of using collaborative patents as a metric by which to evaluate the development trends of collaborative innovation networks^38^. In addition, considering the open and available data sources of patents, their analysis to assess collaborative innovation development is feasible^38^. However, patents have not yet been used to evaluate the structure and relationships of the collaborative innovation network between hospitals and the biomedical industry.

Therefore, it is necessary to determine whether China's supporting policies over the past decade have driven the formation of a collaborative innovation network and to evaluate policy effects by measuring the network through SNA and metrics obtained from jointly owned patents. This study aims to explore the structure of and relationships in the collaborative innovation network between hospitals and the biomedical industry in China over the past decade based on patents by SNA, and examines the roles of the social actors involved. This study also aims to provide a more appropriate method and perspective for elucidating such collaborations and obtaining quantitative evidence to systematically advance the systematic collaborative innovation network between hospitals and the biomedical industry in China and similar countries or regions.

## Results

### Quantitative characteristics of collaborative innovation

In China, the number of patents in the field of biomedicine increased gradually and steadily from 2011 to 2020. The overall trend in the number of patents jointly owned by hospitals and biomedical enterprises generally increased from 2011 to 2020, although there was a slight reduction from 2015 to 2016 and from 2018 to 2019. However, as shown in Table 1, the number of jointly owned patents accounted for a significantly small proportion of all biomedical patents (approximately 0.2%), and this proportion did not exhibit a positive trend.

**Table 1:** Number of biomedical patents in China from 2011 to 2020.

Year	2011	2012	2013	2014	2015	2016	2017	2018	2019	2020
Number of all biomedical patents	8794	11,977	15,508	16,658	19,294	20,932	23,078	25,757	27,162	30,815
Number of biomedical patents jointly owned by hospitals and biomedical enterprises	24	25	29	33	53	47	59	61	57	72
Proportion of jointly owned patents in the number of all biomedical patents	0.27%	0.21%	0.19%	0.20%	0.27%	0.22%	0.26%	0.24%	0.21%	0.23%

### Regional characteristics of collaborative innovation

**Figure 1:**
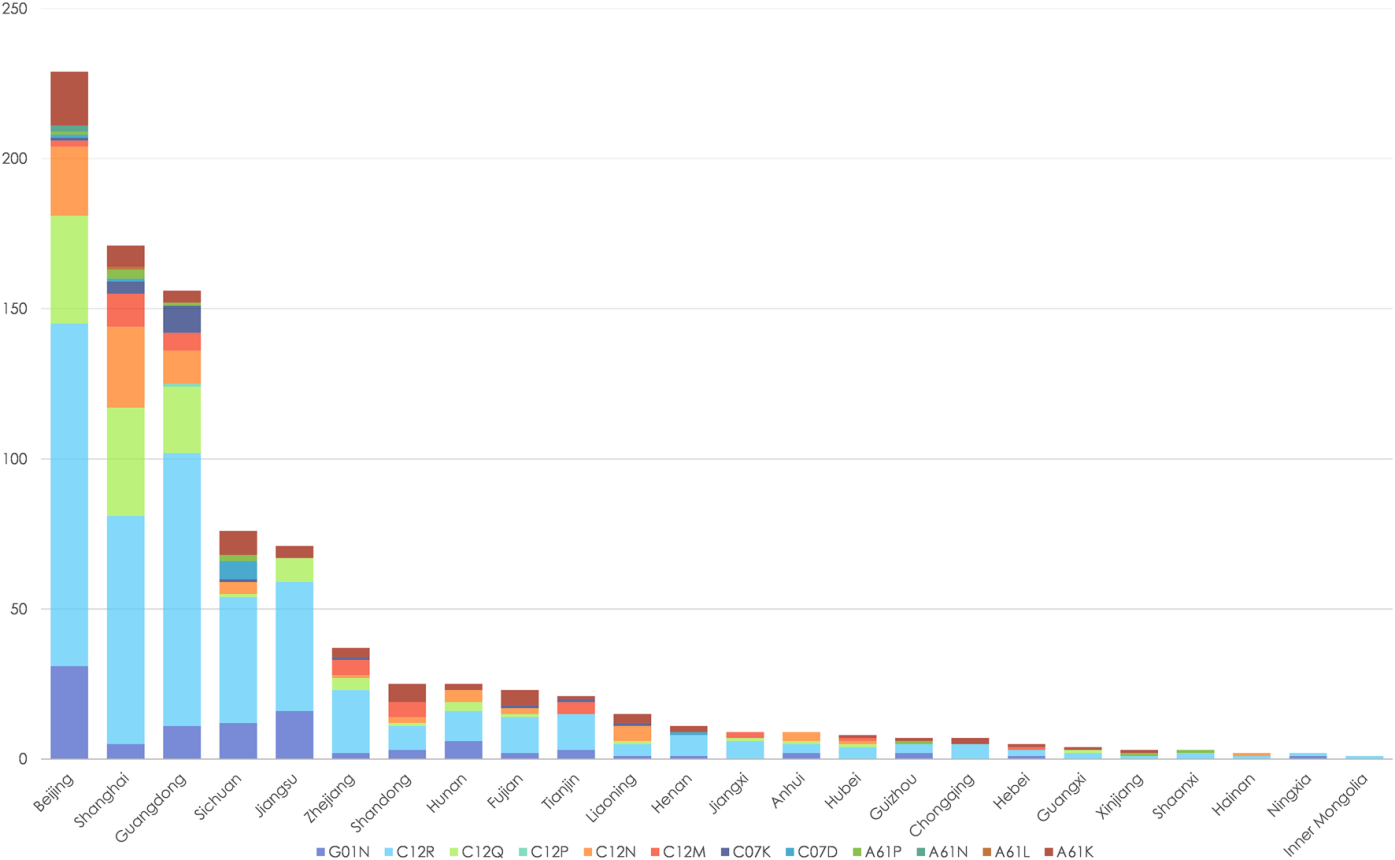
Regional distribution of different types of biomedical patents jointly owned by hospitals and biomedical enterprises in China (2011–2020).

The jointly owned biomedical patent network between hospitals and biomedical enterprises covered 24 regions in mainland China. The top three regions with the highest number of joint biomedical patents were China’s three most famous metropolitan areas: Beijing, Shanghai, and Guangdong (Fig. 1).

**Figure 2:**
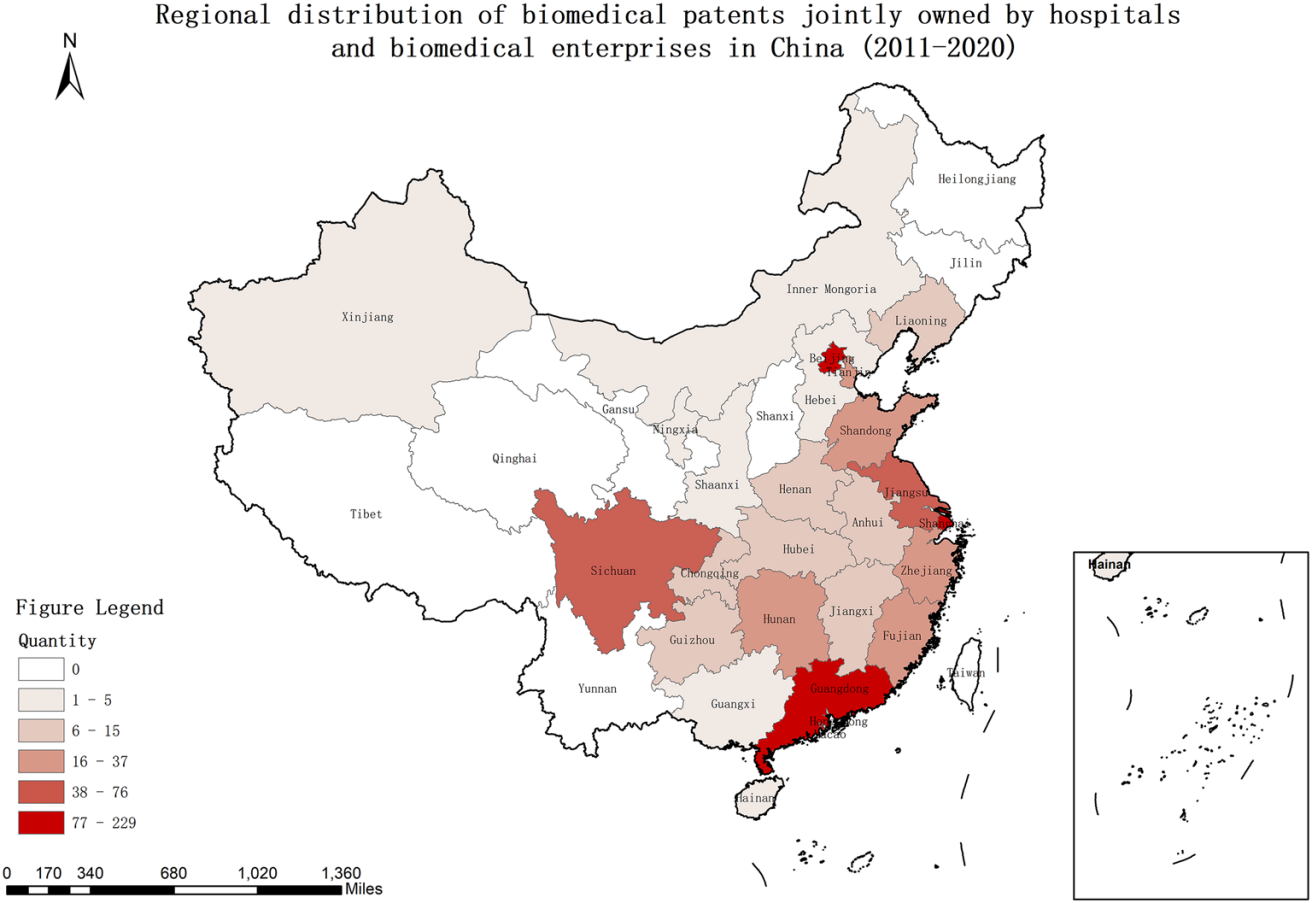
Regional distribution of biomedical patents jointly owned by hospitals and biomedical enterprises in China (2011–2020).

In addition, most collaborative innovation activities of joint biomedical patent applications between hospitals and biomedical enterprises occurred in southeast China. Specifically, according to the number of joint patents, the 24 regions in this network were divided into five levels: 77–229, 38–76, 15–37, 6–15, and 1–5. The largest number (the first level) includes the three regions of Beijing, Shanghai, and Guangdong, indicating the strongest collaborative innovation development. The second level includes the two regions of Sichuan and Jiangsu, suggesting good collaborative innovation development. The third to fifth levels include five, seven, and seven regions, respectively, revealing weak collaborative innovation development status (Fig. 2). Furthermore, to examine whether the number of collaborative innovation activities is correlated with geographical locations of organizations, a quadratic assignment procedure (QAP) was conducted. The QAP result indicated a positive correlation among organizations in northwest China, and a negative correlation among organizations in southeast China and between organizations in northwest and southeast China. This result indicates that collaborative innovation is less likely between organizations in northwest and southeast China and among organizations in southeast China, while organizations in northwest China are more likely to practice collaborative innovation (Table 4).

Furthermore, the most biomedical patents jointly owned by hospitals and biomedical enterprises are distributed in the more economically developed regions. Specifically, according to values of regional gross domestic product (GDP) in 2020^39^, the 24 regions in this network were divided into five ranks:≥ 10, 5–10, 3–5, 1–3, and < 1 trillion CNY. Although there are only 13 regions for ranks 1 to 3, 91.4% of biomedical patents jointly applied and owned by hospitals and biomedical enterprises are in these economically developed regions. Regions that have not yet experienced collaborative innovation activities of joint patent applications (without joint patents) are all in economically undeveloped areas (regions in ranks 4 and 5; Table 2). Additionally, the QAP examined whether the number of collaborative innovation activities is correlated with regional economic development levels. The QAP result suggests a positive correlation among organizations in economically undeveloped areas, a negative correlation between organizations in economically developed and undeveloped areas, and no correlation among organizations in economically developed areas. This result indicates that collaborative innovation is less likely between organizations in economically developed and undeveloped areas, while organizations in economically undeveloped areas are more likely to practice collaborative innovation (Table 4).

**Table 2:** Regional economic characteristic and distribution of biomedical patents jointly owned by hospitals and biomedical enterprises in China (2011–2020).

Region	GDP (100 million CNY)	Ranking	Number of jointly owned biomedical patents		
			N	Percentage (%)	Cumulative percentage (%)
Guangdong	111,151.6	Rank 1	156	17.0	17.0
Jiangsu	102,807.7	Rank 1	71	7.7	24.7
Shandong	72,798.2	Rank 2	25	2.7	27.4
Zhejiang	64,689.1	Rank 2	37	4.0	31.4
Henan	54,259.4	Rank 2	11	1.2	32.6
Sichuan	48,501.6	Rank 3	76	8.3	40.9
Fujian	43,608.6	Rank 3	23	2.5	43.4
Hubei	43,004.5	Rank 3	8	0.9	44.2
Hunan	41,542.6	Rank 3	25	2.7	47.0
Shanghai	38,963.3	Rank 3	171	18.6	65.5
Anhui	38,061.5	Rank 3	9	1.0	66.5
Hebei	36,013.8	Rank 3	5	0.5	67.1
Beijing	35,943.3	Rank 3	229	24.9	92.0
Shanxi	26,014.1	Rank 4	3	0.3	92.3
Jiangxi	25,782.0	Rank 4	9	1.0	93.3
Chongqing	25,041.4	Rank 4	7	0.8	94.0
Liaoning	25,011.4	Rank 4	15	1.6	95.7
Yunnan	24,555.7	Rank 4	0	0.0	95.7
Guangxi	22,120.9	Rank 4	4	0.4	96.1
Guizhou	17,860.4	Rank 4	7	0.8	96.8
Shanxi	17,835.6	Rank 4	0	0.0	96.8
Inner Mongoria	17,258.0	Rank 4	1	0.1	97.0
Tianjin	14,008.0	Rank 4	21	2.3	99.2
Xinjiang	13,800.7	Rank 4	3	0.3	99.6
Heilongjiang	13,633.4	Rank 4	0	0.0	99.6
Jilin	12,256.0	Rank 4	0	0.0	99.6
Gansu	8979.7	Rank 5	0	0.0	99.6
Hainan	5566.2	Rank 5	2	0.2	99.8
Ningxia	3956.3	Rank 5	2	0.2	100.0
Qinghai	3009.8	Rank 5	0	0.0	100.0
Tibet	1902.7	Rank 5	0	0.0	100.0

### Professional field characteristics of collaborative innovation

**Figure 3:**
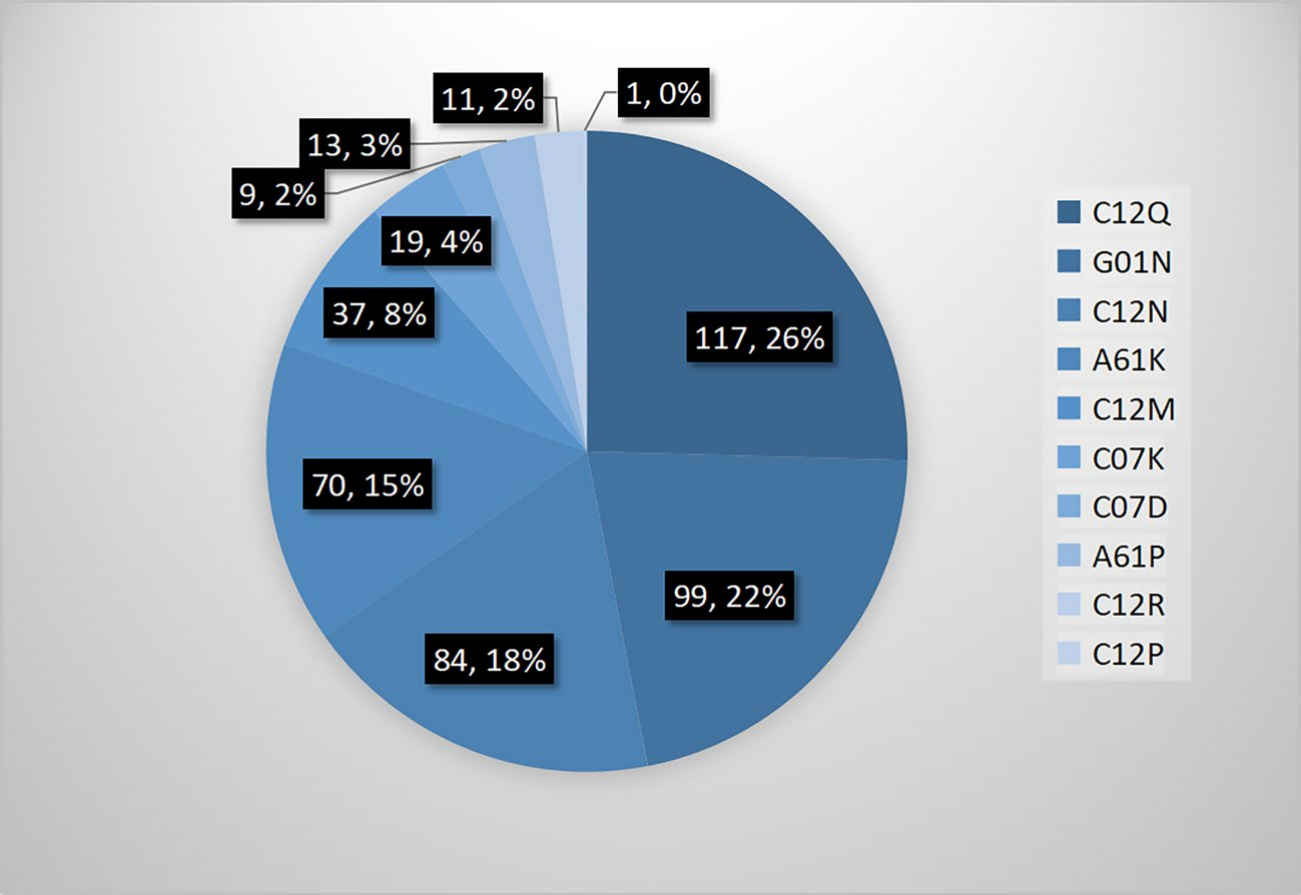
Professional distribution of jointly owned biomedical patents in China from 2011 to 2020.

The top five classifications of biomedical patents jointly owned by hospitals and biomedical enterprises in China were G01N, C12N, A61K, A61P, and C12Q, according to IPC numbers. Patent applications for C12M, C07K, C12R, C12P, and C07D were significantly fewer (Fig. 3).

### Overall network analysis

**Figure 4:**
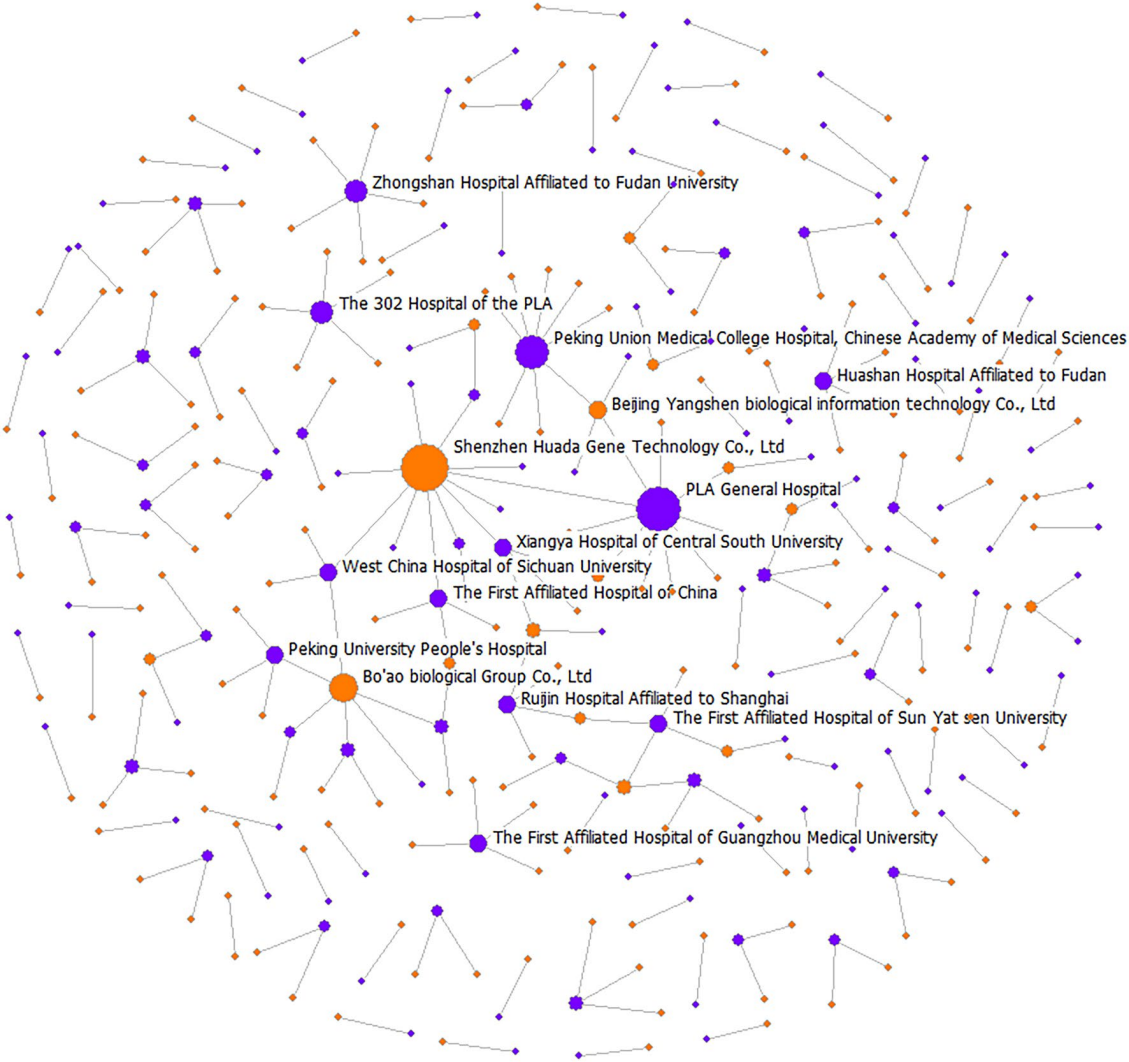
Visual diagram of the collaborative innovation network between hospitals and the biomedical industry.

The collaborative innovation network between hospitals and biomedical enterprises was established based on the overall number of jointly owned patents from 2011 to 2020; the trend over time was not considered. Therefore, this is an undirected one-mode network. It has 341 nodes, which include 144 hospitals, 197 biomedical enterprises, and 460 edges between these nodes, representing joint patent partnership (Fig. 4). The overall network density is 0.004, thereby indicating that the network relationship is at a low concentration level, the overall network structure is sparse, the connectivity is relatively weak, and the degree of resource sharing is not high. Additionally, the collaborative innovation network is not a connected graph. This undirected network has 113 components, and there is a giant component with 70 nodes (Supplementary file). Most nodes at the edge of the network only have one connection, and the overall network is still in the low-frequency collaborative stage.

### Centrality analysis

Degree centrality indicates the most active institutions in the collaborative innovation network. The analysis result shows that the top two institutions with the highest degree centrality are Shenzhen Huada Gene Technology Co., Ltd., and the Beijing Union Medical College Hospital of the Chinese Academy of Medical Sciences, with 11 and 10 partners, respectively (Table 3). The distribution characteristic of degree centrality conforms to the exponential distribution (Kolmogorov–Smirnov test, *P* < 0.001).

**Table 3:** High rankings of degree centrality metric.

Ranking	Patent owner	Region	Institution type	Degree centrality
1	Shenzhen Huada Gene Technology Co., Ltd	Guangdong	Enterprise	11.000
2	PLA General Hospital	Beijing	Hospital	10.000
3	Peking Union Medical College Hospital, Chinese Academy of Medical Sciences	Beijing	Hospital	8.000
4	Bo'ao biological Group Co., Ltd	Shanghai	Enterprise	6.000
5	Zhongshan Hospital Affiliated to Fudan University	Shanghai	Hospital	5.000
6	The 302 Hospital of the PLA	Beijing	Hospital	5.000
7	Beijing Yangshen biological information technology Co., Ltd	Beijing	Enterprise	4.000
8	West China Hospital of Sichuan University	Sichuan	Hospital	4.000
9	Ruijin Hospital Affiliated to Shanghai Jiao Tong University School of Medicine	Shanghai	Hospital	4.000
10	Xiangya Hospital of Central South University	Hunan	Hospital	4.000
11	The First Hospital of China Medical University	Liaoning	Hospital	4.000
12	The First Affiliated Hospital of Sun Yat sen University	Guangdong	Hospital	4.000
13	Peking University People's Hospital	Beijing	Hospital	4.000
14	Huashan Hospital, Fudan University	Shanghai	Hospital	4.000
15	The First Affiliated Hospital of Guangzhou Medical University	Guangdong	Hospital	4.000
16	Tianhao Biomedical Technology (Suzhou) Co., Ltd	Jiangsu	Enterprise	3.000
17	Forevergen Co., Ltd	Guangdong	Enterprise	3.000
18	Children's Hospital of Shanghai	Shanghai	Hospital	3.000
19	The Third People's Hospital of Shenzhen	Guangdong	Hospital	3.000
20	Shanghai Changzheng Hospital	Shanghai	Hospital	3.000
21	The 309th Hospital of the PLA	Beijing	Hospital	3.000
22	Cancer Hospital Chinese Academy of Medical Sciences	Beijing	Hospital	3.000
23	Renji Hospital Affiliated to Shanghai Jiaotong University School of Medicine	Shanghai	Hospital	3.000
24	Sun Yat-sen University Cancer Center	Guangdong	Hospital	3.000
25	Ji’an Central Hospital	Shanghai	Hospital	3.000

In addition, the top 25 institutions with the highest degree centrality are clustered in seven regions, of which six regions are economically developed areas (Table 2). Specifically, most are located in Shanghai (8/25), Beijing (7/25), and Guangdong (6/25); Sichuan, Hunan, Liaoning (economically undeveloped region), and Jiangsu each have one institution (Table 3).

Furthermore, actors are considered big players if the degree centrality is at least 3.000; otherwise, they are conceived as small players. Table 3 shows all 25 big players, of which 80% (20/25) are hospitals, while only 20% are biomedical enterprises (5/25). Moreover, QAP was conducted to examine whether the number of collaborative innovation activities are correlated with the type of players. The QAP result revealed a negative correlation among big players, a positive correlation among small players, and no correlation between big and small players. This result indicates that collaborative innovation is less likely between big players, while small players are more likely to practice collaborative innovation (Table 4).

**Table 4:** Results of QAP.

Attribute features	Expected	Observed	Difference	*P* ≥ Difference	*P* ≤ Difference
Geographical locations					
1 northwest-northwest	125.478	147.000	21.522	0.000	1.000
2 southeast-northwest	88.985	75.000	− 13.985	0.983	0.027
3 southeast-southeast	15.537	8.000	− 7.537	0.991	0.021
Regional economic development levels					
1 underdeveloped-developed	173.224	185.000	11.776	0.010	0.996
2 developed-underdeveloped	52.848	40.000	− 12.848	0.995	0.010
3 developed-developed	3.928	5.000	1.072	0.349	0.807
Type of players					
1 big player-big player	140.892	101.000	− 39.892	1.000	< 0.001
2 big player-small player	78.391	80.000	1.609	0.425	0.628
3 small player-small player	10.716	49.000	38.284	< 0.001	1.000

## Discussion

The collaborative innovation network from 2011 to 2020 based on jointly owned biomedical patents in mainland China included 144 hospitals and 197 biomedical enterprises, and involved 460 connections. Although the number of biomedical patents jointly owned by hospitals and biomedical enterprises showed an increasing trend, the total amount and its proportion of all biomedical patents were still small, especially in less-developed regions; collaborative innovation is more likely between organizations in less-developed regions while collaborative innovation between developed and less-developed regions is less likely. This jointly owned biomedical patent network comprised 24 regions in mainland China and presented obvious regional characteristics. Most biomedical patents jointly owned by hospitals and biomedical enterprises were primarily distributed in southeast China, especially in China’s three most famous metropolitan areas: Beijing, Shanghai, and Guangdong; collaborative innovation is more likely between organizations in northwest China, while collaborative innovation between organizations in southeast China or between organizations in southeast and northwest China is less likely. Patents related to the G01N, C12N, and A61K classifications were the most popular for ensuring collaborative innovation. The overall network is an undirected one-mode network and not a connected graph. This network is sparse with a density of 0.004, and has 113 components with a giant component including 70 nodes. A biomedical enterprise in Guangdong and a hospital in Beijing have the highest degree centrality, and actors with higher degree centrality are mainly hospitals and clustered across six economically developed regions. There are 25 big players in the network; however, collaborative innovation is less likely between these big players and collaborative innovation between small players is more likely. Overall, after experiencing ten-year collaborative innovation accelerating policy efforts in China, from the perspective of joint patents, the collaborative innovation network between hospitals and biomedical enterprises is still very sparse and without good connectivity.

This study used collaborative patent statistics to observe and assess collaborative innovation networks between hospitals and biomedical industry for the first time, which clearly indicated China’s policy effects over the past decade. The usage of collaborative patents provided a feasible and reasonable method and perspective by which to quantitatively elucidate collaborative innovation networks in the biomedical field. The characteristics, structure, relationships, and roles of innovation entities can be comprehensively analyzed. Moreover, the collaborative innovation network established in this study provides the following evidence by which to strengthen the existing network and develop new collaborations.

Although the level of collaborative innovation between hospitals and the biomedical industry in China remains low, the attention paid to collaborative innovation keeps increasing. The overall quantitative tendency in joint patents continues to increase. Nonetheless, jointly owned patents were far fewer than the total in the field of biomedicine, for the following reasons. First, in China, there is a large gap in collaborative innovation between hospitals and the biomedical industry between various regions with different levels of economic development. Our findings are consistent with existing evidence that the number of patents might be correlated with the level of regional economic development^40^. Our results show that the number of collaborative patents is fewer in both the less-developed regions and the northwest China, and the two findings are essentially consistent; that is, the number of patents jointly owned by hospitals and biomedical enterprises is much lower in the economically less-developed regions of China. However, it is important to note that although the current number is low, hospitals and biomedical enterprises in economically underdeveloped regions and northwest China are more motivated to implement collaborative innovation. Therefore, investments or policy support should be guaranteed in these regions to create a better environment for collaborative innovation between hospitals and the biomedical industry^22^. Second, research statistics suggest that the transformation rate of hospitals, even in developed regions of China, such as Shanghai, is only 3%, which is considerably lower than the international transformation rate of approximately 30–50%^41^. Furthermore, low transformation rates are associated with low densities and frequencies of collaborative innovation networks between hospitals and the biomedical industry^41^. Third, evidence shows that in China, only 5% of primary drugs are available among all approved biological drugs^42^. Investments from biomedical enterprises aiming to fund innovative research are significantly low, and this attribute hinders the actualization of collaborative innovation between hospitals and the biomedical industry. However, such investments must satisfy the requirements associated with both the scale and capital strength of biomedical enterprises. According to a study conducted in the United States, the median research and development investment for a new drug was $985.3 million, and the mean investment was $1.336 billion^43^. Moreover, over recent years, the levels of investment required to ensure innovative research from both public and private sources keep increasing^44^, thereby suggesting the potential for creating collaborative innovation networks between hospitals and biomedical enterprises.

According to IPC analysis, the most jointly owned biomedical patents in China are those involving the G01N (investigating or analyzing materials by determining their chemical or physical properties), C12N (microorganisms or enzymes; compositions thereof; propagating, preserving, or maintaining microorganisms; mutation or genetic engineering; culture media), A61K (preparations for medical, dental, or toilet purposes), A61P (specific therapeutic activity of chemical compounds or medicinal preparations), and C12Q (measuring or testing processes involving enzymes, nucleic acids or microorganisms; compositions or test papers thereof; processes of preparing such compositions; condition-responsive control in microbiological or enzymological processes) classifications. These types of patents are generally in accordance with popular trends reported in other studies^45^. Identifying such trends can not only suggest the future professional direction of collaborative innovation in China^46^, but guide policy-makers to prioritize resources and partnerships to strengthen the existing collaborative innovation network and promote new collaborations^20^.

Considering that most of the actors with higher degree centrality are hospitals, more hospitals are active in practicing collaborative innovation activities than the biomedical industry. This aspect may be related to the gap in the development stages of hospitals and biomedical enterprises. Owing to the concepts and practices employed in research hospitals^47^, hospitals in China have experienced rapid development over the past two decades^48^, and this has been a national medium and long-term development plan^49^. Since the main task of research hospitals is conducting innovative research and collaborative translation^50,51^, hospitals have stronger initiatives for seeking and actualizing collaborative innovation^52^. However, similar practices and policy directions were not proposed in the field of biomedicine until a decade ago^14^. This attribute explains the delayed development in the collaborative innovation of biomedical enterprises compared with hospitals. Furthermore, the whole network is still sparse and without good connectivity, thereby indicating that the collaborative innovation capabilities of both hospitals and biomedical enterprises are very weak and far from facilitating the development of a collaborative innovation network for breaking through the current elementary stage^53^. It is worth noting that small players are more likely to practice collaborative innovation, suggesting a direction to increase the connectivity of the network by identifying potential small players and providing more policy support and initiatives for them.

There are several limitations associated with this study. First, considering that the scope of the IPC in the field of biomedicine varies across different studies, the biomedical patents included in this study are based on a broader range, which can be made highly accurate in future studies. Second, patent data were obtained from only IncoPat. The sources of patent data should be increased in future studies. Third, we selected the first two applicants if there were three or more applicants, thereby neglecting some actors who made small contributions to the collaborative innovation network and some new relationships. Fourth, although it is reasonable to evaluate the levels of collaborative innovation using the metric of patents jointly owned by hospitals and biomedical enterprises, a comprehensive measurement metric including aspects other than patents can be used to better reflect collaborative innovation levels in future studies. Fifth, although the aim of this study was to examine whether the ten-year efforts on collaborative innovation of China’s supporting policies have driven the network formation and analyze its in-depth characteristics, studying the network's evolution over time could be interesting and valuable. However, such changes have not been considered currently, which should be included in future studies.

## Conclusions

This study describes and analyzes a decade-long collaborative innovation network structure and relationships between hospitals and the biomedical industry based on jointly owned patents. Policy support and increased investment strategy should be encouraged to improve collaborative innovation density and frequency, especially in less-developed and northwest regions. The government should further enhance the quality and quantity of collaborative innovation by stimulating the collaborative innovation initiatives of hospitals and inspiring the potential of developing more biomedical enterprises. To break through the dispersed and low connectivity limitation, it is necessary to strengthen the existing collaborative innovation network, and promote new collaborations. Policy-makers should strategically help small players establish more partnerships and big players improve collaborative innovation capabilities.

## Methods

### Study design

Owing to rapid advancements in the fields of medicine and biomedicine in China over the past decade, the collaborative innovation network between hospitals and the biomedical industry in China was constructed based on patents jointly owned by hospitals and biomedical enterprises from 2011 to 2020. The adjacency matrix of patent applicants was constructed using Excel 16.16.27. The generated matrix was imported into UCINET 6.732 (Harvard: Analytic Technologies) to generate the network topology of the collaborative innovation network and analyze the network’s cohesion and centrality metrics^54^.

### Data source

The patent data jointly owned by hospitals and biomedical enterprises were collected from the IncoPat scientific and technological innovation intelligence platform (BEIJING INCOPAT CO., LTD.). Patents with biomedical-related codes were extracted according to the International Patent Classification (IPC) system. The extraction process was also based on keywords obtained from existing studies to ensure comprehensiveness.

The inclusion criteria involved the following aspects: (1) patents were jointly applied and authorized by at least one hospital and one biomedical enterprise; (2) the application was in mainland China; (3) the patent type was a valid patent for invention; (4) the application time was from January 1, 2011 to December 31, 2020; (5) IPC codes were A61P (special therapeutic activity of chemical compounds or medicinal preparations), A61K (preparations for medical, dental, or toilet purposes), C07K (peptides), C07H (sugars; derivatives thereof; nucleosides; nucleotides; nucleic acids), C12N (microorganisms or enzymes; compositions thereof; propagating, preserving, or maintaining microorganisms; mutation or genetic engineering; culture media), C12M (apparatus for enzymology or microbiology), C12P (fermentation or enzyme-using processes to synthesize a desired chemical compound or composition or separate optical isomers from a racemic mixture), C12Q (measuring or testing processes involving enzymes, nucleic acids or microorganisms; compositions or test papers thereof; processes for preparing such compositions; condition-responsive control in microbiological or enzymological processes), and G01N (investigating or analyzing materials by determining their chemical or physical properties); (6) keywords were searched, including cell, gene, interferon, interleukin, hormone, recombination, protein, enzyme, antibody, monoclonal antibody, antigen, receptor, fermentation, nucleic acid, amino acids, nucleotides, peptides, serum, coagulants, diagnostic reagents, inhibitors, cross-linking agents, vaccines, vitamins, traditional Chinese medicine, and equipment; (7) the first two applicants were included if there were three or more applicants for a patent; and (8) patent application regions included the regions of the first two applicants. The exclusion criteria were: (1) repeatedly applied patents; (2) patents not related to the field of biomedicine; (3) patents applied by organizations and individuals from other countries; (4) patents owned by individuals; and (5) patents owned only by hospitals or biomedical enterprises.

### Network visualization and metrics

The analysis of the collaborative innovation network includes visualization and quantitative metrics. In terms of visualization, the actor in the network is usually represented as a circle or square, and its size in this network reflects the number of patents. The lines between two actors represent the collaborative innovation between them.

Quantitative metrics can be divided into metrics at the network and individual levels. Metrics at the network level include the number of nodes, the number of edges, density, and components. The number of nodes refers to the number of all patent-owning institutions included in the network. The number of edges refers to the number of connections between nodes, which is the total number of cooperative relationships between patent owners. Network density is an index for evaluating the internal information connectivity of the network, which reveals the closeness between the collaborative innovation network and the collaborative relationship of biomedicine in China^55^. Component analysis can be used to test the connectivity of the network and identify the giant component.

In terms of metrics at the individual level, centrality measures are the most common in SNA. Because this network is not a connected graph, only degree centrality holds significance. Degree centrality is used to measure the degree of communication between a node and other nodes in a network. If a node has a high number of links, it is in a highly important and central position in a network^56^. QAP is used to explore the correlation between attribute features and the collaborative innovation relationship.

### Supplementary Information

Supplementary Table S1.

## Data Availability

The datasets generated and analyzed during the current study are available from the corresponding author on reasonable request.
