# Supplementary material for: Social network analysis of a decade-long collaborative innovation network between hospitals and the biomedical industry in China
Source: Sci Rep. 2024 May 18;14:11374. doi: 10.1038/s41598-024-62082-3 (PMC11102486; doi:10.1038/s41598-024-62082-3)
Supplement: Supplementary file 1 — Supplementary Table S1. [file 41598_2024_62082_MOESM1_ESM.docx]

**Supplementary Table S1. The Connected Components of biomedical patents jointly owned by hospitals and biomedical enterprises in China**

| **Component** | **Nodes** | **Proportion** |
| --- | --- | --- |
| 1 | 70 | 0.205 |
| 2 | 5 | 0.015 |
| 3 | 2 | 0.006 |
| 4 | 2 | 0.006 |
| 5 | 3 | 0.009 |
| 6 | 4 | 0.012 |
| 7 | 2 | 0.006 |
| 8 | 2 | 0.006 |
| 9 | 3 | 0.009 |
| 10 | 3 | 0.009 |
| 11 | 3 | 0.009 |
| 12 | 6 | 0.018 |
| 13 | 3 | 0.009 |
| 14 | 2 | 0.006 |
| 15 | 2 | 0.006 |
| 16 | 3 | 0.009 |
| 17 | 4 | 0.012 |
| 18 | 2 | 0.006 |
| 19 | 2 | 0.006 |
| 20 | 3 | 0.009 |
| 21 | 3 | 0.009 |
| 22 | 5 | 0.015 |
| 23 | 2 | 0.006 |
| 24 | 2 | 0.006 |
| 25 | 2 | 0.006 |
| 26 | 3 | 0.009 |
| 27 | 2 | 0.006 |
| 28 | 2 | 0.006 |
| 29 | 2 | 0.006 |
| 30 | 6 | 0.018 |
| 31 | 2 | 0.006 |
| 32 | 3 | 0.009 |
| 33 | 2 | 0.006 |
| 34 | 2 | 0.006 |
| 35 | 5 | 0.015 |
| 36 | 2 | 0.006 |
| 37 | 2 | 0.006 |
| 38 | 2 | 0.006 |
| 39 | 3 | 0.009 |
| 40 | 2 | 0.006 |
| 41 | 2 | 0.006 |
| 42 | 2 | 0.006 |
| 43 | 3 | 0.009 |
| 44 | 2 | 0.006 |
| 45 | 2 | 0.006 |
| 46 | 2 | 0.006 |
| 47 | 4 | 0.012 |
| 48 | 2 | 0.006 |
| 49 | 2 | 0.006 |
| 50 | 2 | 0.006 |
| 51 | 4 | 0.012 |
| 52 | 2 | 0.006 |
| 53 | 3 | 0.009 |
| 54 | 3 | 0.009 |
| 55 | 2 | 0.006 |
| 56 | 3 | 0.009 |
| 57 | 2 | 0.006 |
| 58 | 2 | 0.006 |
| 59 | 4 | 0.012 |
| 60 | 2 | 0.006 |
| 61 | 3 | 0.009 |
| 62 | 2 | 0.006 |
| 63 | 2 | 0.006 |
| 64 | 2 | 0.006 |
| 65 | 2 | 0.006 |
| 66 | 2 | 0.006 |
| 67 | 2 | 0.006 |
| 68 | 2 | 0.006 |
| 69 | 2 | 0.006 |
| 70 | 3 | 0. 009 |
| 71 | 3 | 0.009 |
| 72 | 2 | 0.006 |
| 73 | 2 | 0.006 |
| 74 | 2 | 0.006 |
| 75 | 2 | 0.006 |
| 76 | 2 | 0.006 |
| 77 | 2 | 0.006 |
| 78 | 2 | 0.006 |
| 79 | 2 | 0.006 |
| 80 | 3 | 0.009 |
| 81 | 2 | 0.006 |
| 82 | 2 | 0.006 |
| 83 | 2 | 0.006 |
| 84 | 2 | 0.006 |
| 85 | 3 | 0.009 |
| 86 | 2 | 0.006 |
| 87 | 2 | 0.006 |
| 88 | 2 | 0.006 |
| 89 | 2 | 0.006 |
| 90 | 2 | 0.006 |
| 91 | 2 | 0.006 |
| 92 | 2 | 0.006 |
| 93 | 2 | 0.006 |
| 94 | 2 | 0.006 |
| 95 | 2 | 0.006 |
| 96 | 2 | 0.006 |
| 97 | 2 | 0.006 |
| 98 | 2 | 0.006 |
| 99 | 2 | 0.006 |
| 100 | 2 | 0.006 |
| 101 | 2 | 0.006 |
| 102 | 2 | 0.006 |
| 103 | 2 | 0.006 |
| 104 | 2 | 0.006 |
| 105 | 2 | 0.006 |
| 106 | 2 | 0.006 |
| 107 | 2 | 0.006 |
| 108 | 2 | 0.006 |
| 109 | 2 | 0.006 |
| 110 | 2 | 0.006 |
| 111 | 2 | 0.006 |
| 112 | 2 | 0.006 |
| 113 | 2 | 0.006 |
